# Genome-wide studies of telomere biology in budding yeast

**DOI:** 10.15698/mic2014.01.132

**Published:** 2014-03-01

**Authors:** Yaniv Harari, Martin Kupiec

**Affiliations:** 1 Department of Molecular Microbiology and Biotechnology, Tel Aviv University, Ramat Aviv 69978, Israel.

**Keywords:** yeast, telomeres, genome stability, cancer, aging, systems biology

## Abstract

Telomeres are specialized DNA-protein structures at the ends of eukaryotic chromosomes. Telomeres are essential for chromosomal stability and integrity, as they prevent chromosome ends from being recognized as double strand breaks. In rapidly proliferating cells, telomeric DNA is synthesized by the enzyme telomerase, which copies a short template sequence within its own RNA moiety, thus helping to solve the “end-replication problem”, in which information is lost at the ends of chromosomes with each DNA replication cycle. The basic mechanisms of telomere length, structure and function maintenance are conserved among eukaryotes. Studies in the yeast *Saccharomyces cerevisiae* have been instrumental in deciphering the basic aspects of telomere biology. In the last decade, technical advances, such as the availability of mutant collections, have allowed carrying out systematic genome-wide screens for mutants affecting various aspects of telomere biology. In this review we summarize these efforts, and the insights that this Systems Biology approach has produced so far.

## INTRODUCTION

The ends of the eukaryotic chromosomes are protected by special nucleoprotein structures called telomeres. Telomeres play pivotal roles in maintaining the stability of the genome: they serve to distinguish between the natural chromosomal ends, which should not be repaired, and double stranded DNA breaks (DSBs), which need to be repaired urgently to prevent loss of genomic information. DSBs occur often, due to external insults, or to the normal metabolism of the cell 1. Telomeres protect the chromosomal ends by virtue of the special chromatin configuration conferred by telomeric proteins, as well as by particular three-dimensional folding of the DNA 2,3. In addition, telomeres provide a solution to the “end-replication problem”: due to the need for primers, the regular DNA polymerases are unable to fully replicate the chromosomal ends 4,5; this problem is exacerbated by resection of the ends by nucleases, which leads to loss of information from the chromosomal ends in each cell division. Eventually most somatic cells senesce and stop dividing 6,7,8.

Unicellular organisms and embryonic mammalian cells, which are highly proliferative, solve the end-replication problem by expressing telomerase 9,10, a specialized reverse transcriptase able to extend the telomeres by copying telomeric sequences from an internal RNA template. Telomerase is not expressed in most somatic cells; strikingly, it suffices to express a functional and active telomerase to overcome cellular senescence in these cells 11. Cancer cells are also highly proliferative, and therefore require functional telomeres: in about 80% of tumors, the telomerase gene is expressed 12; in the rest, an alternative mechanism, based on homologous recombination, allows telomere length extension (*a*lternative *l*engthening of *t*elomeres or ALT; reviewed in 13). Moreover, replenishing telomeres is one of the few essential steps that a normal mammalian fibroblast must take in order to become cancerous 14. Mutations that affect telomere function result in human diseases, such as Idiopathic Pulmonary Fibrosis, Dyskeratosis Congenita, and others 15,16,17. Thus, our understanding of the biology of telomeres has significant medical implications, in particular for the fields of aging and cancer.

Although differences exist in the composition and organization of telomeres in yeast and mammals, many basic rules are universal. In 2009 Elizabeth Blackburn, Carol Greider and Jack Szostak received the Nobel Prize in Medicine for their work on telomeres and telomerase. Much of this work was carried out in model organisms, including the yeast *Saccharomyces cerevisiae*. Other yeasts, particularly *K. lactis *and *S. pombe*, have also contributed extensively to our understanding of telomere biology. In this review we will concentrate particularly on *S. cerevisiae.*

## COMPOSITION OF YEAST TELOMERES 

**Figure 1:**
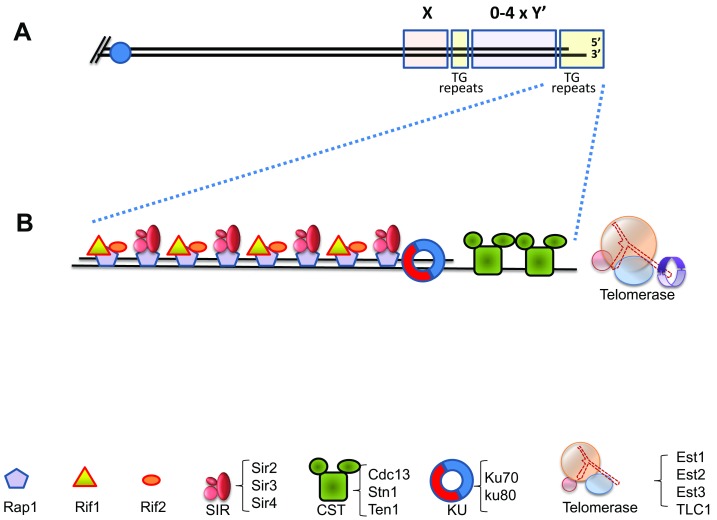
FIGURE 1: Structure of the yeast telomere. **(A)** Schematic representation of a yeast telomere. All yeast chromosomes carry subtelomeric repeats called X elements, and between 0 and 4 copies of another sub-telomeric element, the Y’ sequences. Telomeric repeats are composed of variations of the T(G1-3) formula. The TG-rich strand (with its 3’ OH) is longer than the complementary strand (TG overhangs). **(B)** Schematic representation of the telomeric chromatin, with representative proteins. Rap1 binds the telomeric repeats, and Rif1, Rif2 and the SIR proteins bind to Rap1. The Ku heterodimer binds to telomeric dsDNA and the CST complex binds the terminal ssDNA end. Telomerase is recruited to telomeres present in an “extensible” configuration by interactions with the CST.

The basic structure of the yeast telomere is depicted in Figure 1. The RNA template of telomerase (encoded by the *TLC1* gene) bears the template sequence CACACACCCACACCAC 18. However, the telomeric sequence in *S. cerevisiae* is not regular, and can be described as T(G_1-3_) 19,20. Thus, only very short stretches are copied in each round of telomerase activity from the RNA template 21. This contrasts with the sequence regularity observed in other organisms, such as *K. lactis *22.

A number of proteins bind yeast telomeres:

**Rap1: **Rap1, an essential DNA binding protein, recognizes and binds to the yeast telomeric DNA repeats, and serves to determine telomere length by a “counting” mechanism that monitors Rap1 concentration (Figure 1B) 23,24,25. Rap1 also contributes to telomere capping and prevents repair of the telomeres as regular double-stranded breaks, as well as playing roles in localizing telomeres to the nuclear periphery 26,27,28. In addition to its telomeric role, Rap1 also works as a general cellular transcriptional activator and repressor that binds to upstream promoter regions at a large number of genes and interacts with various regulatory proteins 29,30.

**Rif1 and Rif2: **Two proteins, Rif1 and Rif2, bind the C-terminal region of Rap1 (Figure 1B) 31,32. Cells mutated for *RIF1 *or *RIF2 *exhibit long telomeres, indicating that the function of these proteins is to negatively regulate the elongation of telomeres 31,32. Rif1 and Rif2 binding confers to Rap1-bound telomeric DNA a higher order structure by interconnecting different Rap1 units. The functional importance of this structure, and its role in telomere biology are still unclear 33. Despite the coordinated binding, Rif1 and Rif2 seem to work separately of each other. As explained above, *rif1* and *rif2* mutants show elongated telomeres. The double mutant, however, exhibits much longer and unregulated telomeres, indicating that the two proteins participate in alternative regulatory mechanisms 32,34.

**Yku70 and Yku80: **Ku is a conserved complex composed of two proteins of ~70 and ~85 kDa (Yku70 and Yku80 in yeast). It plays central roles in DSB repair by non-homologous end joining (NHEJ), a mechanism in which the broken ends are ligated together irrespectively of their sequence 35. Since NHEJ must be avoided at telomeres (to prevent fusions between chromatids or chromosomes), it is surprising that Ku is also a natural component of telomeres. However, Ku plays an essential role in telomere maintenance (Figure 1B, reviewed in 1). The Ku complex is associated with telomerase RNA (TLC1) and participates in the import of TLC1 to the nucleus 36, and possibly in the recruitment of telomerase 37,38,39. Moreover, the Ku heterodimer helps in anchoring the telomeres to the perinuclear space 28 by a still mysterious mechanism that involves the small protein modifier SUMO 40. Finally, Ku presence seems to prevent exonucleolytic activity at broken chromosomes and at telomeres 41,42,43. Thus, Ku affects almost all aspects of telomere biology, although it is not completely essential for life. Interestingly, specific mutations have been found, which separate the roles that Ku plays in NHEJ and in telomere biology 44,45.

**The CST complex:** Another conserved complex is composed of the Cdc13, Stn1 and Ten1 proteins. This complex structurally resembles Replication Protein A (RPA), which binds ssDNA during cellular DNA replication and DNA repair (reviewed in 46). The CST binds single-stranded telomeric repeats through OB folds, a common motif in ssDNA and RNA binding proteins (Figure 1B) 47. It has been proposed that the CST out-competes and replaces RPA at telomeres; however, RPA can also be detected at telomeres, and is probably functional during DNA replication 48,49,50. Thus, a division of work between the CST and RPA must exist, which is probably intricately linked to the mechanism of replication of telomeres. Stn1 and Ten1, the two proteins associated with Cdc13, were isolated as genetic and physical interactors of Cdc13 51,52. The interactions between these proteins are complex: Stn1 and Ten1 appear to regulate the activity of Cdc13 46; on the other hand, mutations in *STN1* 52 or overexpression of both Stn1 and Ten1 can suppress the lethality of the temperature-sensitive *cdc13-1* mutants 53,54. These results indicate the existence of activities of Stn1 and Ten1 that are carried out independently of Cdc13.

**The SIR complex:** Due to the heterochromatic nature of telomeres, in many organisms genes located close to telomeres undergo silencing (also called *t*elomere *p*osition *e*ffect or TPE). This silencing depends on the Silent Information Regulator (SIR) complex, which consists of three proteins, Sir2, Sir3 and Sir4 (Figure 1B) 55. The complex is recruited to telomeres by interactions with Rap1 and plays also a role in tethering telomeres to the nuclear envelope.

**Telomerase:** Yeast telomerase consists of three proteins, Est1-3 and an RNA molecule encoded by the TLC1 gene; Est2 contains the catalytic activity 56. The name “Est” comes from genetic screens that originally identified “*e*ver *s*horter *t*elomere” mutants, defective in telomerase activity 8,57. The exact role of Est1 and Est3 in telomerase function is still not completely understood. In strains carrying a fusion between Cdc13 and the Est2 catalytic subunit, the essential Est1 subunit becomes dispensable, demonstrating that telomerase is recruited by the Cdc13-Est1 interaction, and this recruitment seems to be the only essential function of Est1 58,59. However, recruitment of Est2 cannot be the sole function of Est1 since mutant Est1 proteins, that retain association with the telomerase enzyme, still affect telomere length 60. In addition, Est1 also favors telomerase-mediated DNA extension *in vitro *through a direct contact with Est2 61.

## ROLES OF TELOMERES IN END PROTECTION AND GENOME REPLICATION

One of the main functions of the telomere is to prevent the cell from repairing its natural chromosomal ends as if they were chromosomal double-stranded breaks (DSBs). This function, called telomere capping, is extremely important: DSBs are among the most serious types of DNA damage a cell can undergo, and efficient response mechanisms have evolved to cope with the presence of even a single DSB. However, attempts by the cells to repair natural telomeres as if they were broken chromosomes will lead to chromatid or chromosomal fusions, starting a cycle of fusions and breakages that will increase genome instability, and thus cell death and/or cancer development 1,62.

If telomeres become uncapped, the cells recognize the presence of broken DNA ends, and react by eliciting the DNA Damage Response (DDR), which in yeast is controlled by the Tel1 and Mec1 protein kinases (orthologs of mammalian ATM and ATR). This response includes cell cycle arrest, and attempts to repair the damage (reviewed in 1). Indeed, when telomeres become uncapped, the cells attempt repair of the exposed DNA ends; a robust resection of the telomeric DNA by nucleases can be observed, as well as telomere-telomere fusions 26,63,64,65. The genetic control of resection differs from the one observed at nontelomeric DSBs: the MRX complex, which usually participates in the initiation of resection, inhibits resection at telomeres, and mutations in the MRX genes exhibit increased ssDNA levels (Foster *et al.*, 2006). An elegant labeling experiment demonstrated that MRX binds specifically to leading-strand telomeres, where it could generate ssDNA for the CST to bind 66.

Telomere uncapping may result as a consequence of inactivation of CST components 63, or of the Ku heteroduplex 42,67. Similarly to the CST and Ku complexes, the Rap1-Rif1-Rif2 complex plays a role in telomere capping. However, Rap1 inactivation leads to cell cycle arrest at G1 instead of the Mec1-dependent G2 arrest 68.

**Figure 2:**
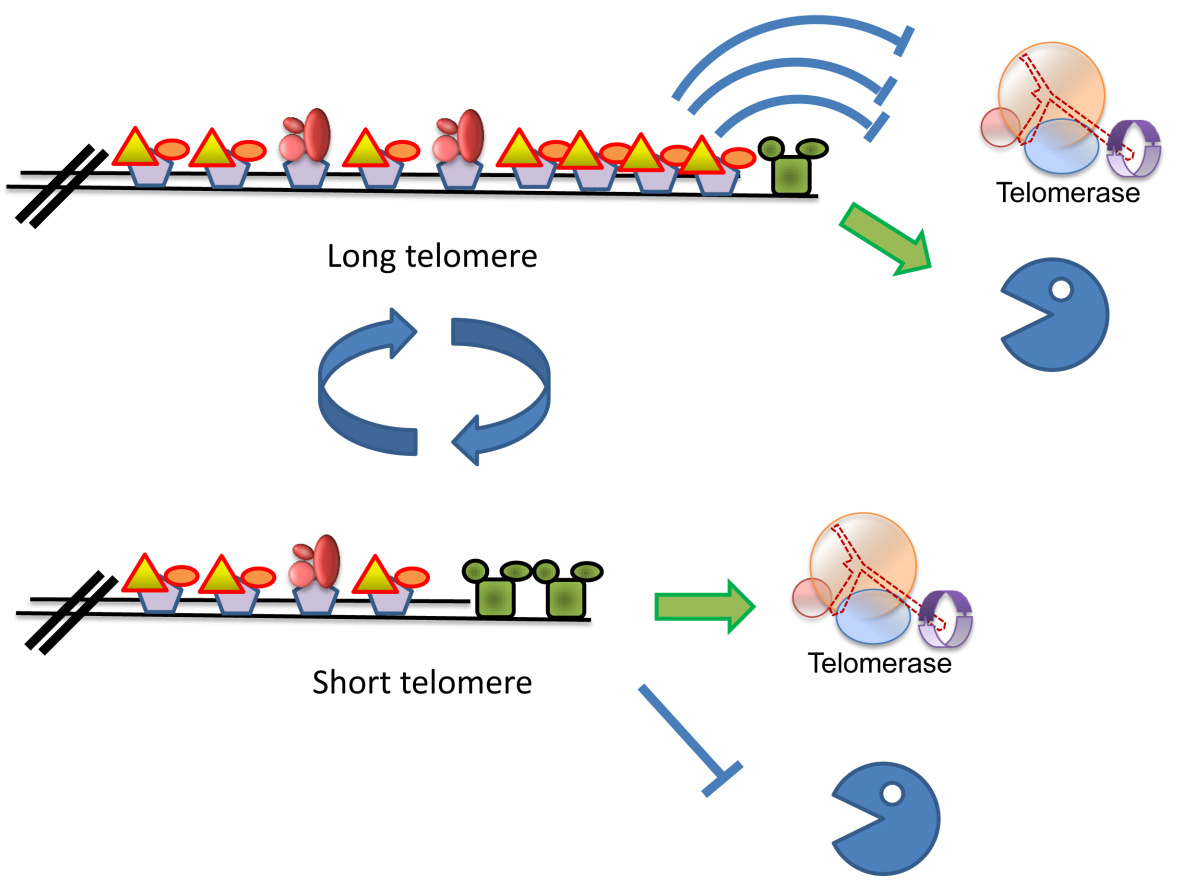
FIGURE 2: Schematic model of the mechanism(s) that keep telomere length homeostasis. Long telomeres carry many copies of the Rap1 protein, and its associated Rif1 and Rif2 proteins, which prevent recruitment of telomerase. Telomeres in this state are said to be in a “non-extendable” configuration 69. Telomeres shorten as a consequence of the “end replication problem” or by the action of exonucleases (Pacman). It is still unclear what circumstances allow recruitment of nucleases. Short telomeres carry a low number of Rap1/Rif units, and the CST is able to recruit telomerase, thus elongating the telomere. It is assumed that the presence of an active telomerase prevents recruitment of shortening nucleases.

As explained above, telomerase plays an important role in solving the “end-replication problem” 4,5. Telomerase is recruited to short chromosomes, which are said to be in an “extensible” configuration 69 (Figure 2). The molecular determinant of this state is still unclear, and may represent a particular protein configuration, a particular state of the DNA/chromatin, or be related to the tethering of telomeres to the nuclear envelope. Telomerase activity must be somehow coordinated with the replication of the rest of the genome. Replication origins located close to telomeres replicate very late in the S-phase of the cell cycle 70; curiously, this phenomenon is independent of the replication origin sequence: any origin located near telomeres is fired late, by a still enigmatic mechanism 71.

The coordination between telomere and genomic replication is complex, and not completely understood. Telomerase is recruited to the shortest telomeres by a mechanism that is regulated by Tel1 (ATM) 72. The level of Cdc13, Est1 and Est3 increases at these telomeres by the end of S-phase, consistent with a model in which telomerase is recruited by interactions between Est1 and the CST 59. Surprisingly, the catalytic subunit, Est2, is present at the telomerase throughout the cell cycle; the significance of this fact is still not understood. The Est1 telomeric subunit becomes dispensable in strains carrying a fusion between Est2 and Cdc13, suggesting that its main function is to recruit telomerase 73. The recruitment of Est3 to telomerase was shown to be Est1-dependent 58. However, expression of Est1 in G1, which results in Est1 and Est3 recruitment to the Est2-TLC1 complex already present at telomeres, does not allow telomerase activation, indicating that additional conditions must be met for telomerase activation 74. These could be CDK1-related (e.g., phosphorylation of one of the proteins by the activated CDK1 may be a pre-requisite), or the telomeric DNA may need to be in a particular molecular configuration for telomerase to act 69. Interestingly, the Rif1 and Rif2 proteins and the Ku heteroduplex may also restrict G1 activation 42,75,76.

## THE SYSTEMS BIOLOGY REVOLUTION

In the last fifteen years, the efforts to get more systematic and complete information about the interactions between molecules resulted in the development of methodologies, such as the DNA microarray, which provide data about the status of the whole genome. This technical achievement lead to a change in attitude, which allowed turning the traditional reductionist approach of Molecular Biology upside-down, to attempt a whole-encompassing view of the cell. One could thus describe genetic or protein interactions in the same way an astronomer would describe relationships between stars in the sky. By analyzing large amounts of data one could discern the existence of order in the distribution of the bodies analyzed (e.g., galaxies in the sky or protein complexes in a cell), and attempt to decipher the rules behind their behavior. In addition, there are “emerging properties”, which cannot be seen by looking at each gene/protein separately and require a “bird’s eye view” to be discerned (as one can see a pattern in the distribution of highways in a map, that are hard to see while traveling a highway by car). A flurry of genome-wide methodologies was launched, in which a systematic approach was taken to try to map all the genes (genomics), RNA molecules (transcriptomics), proteins (proteomics), and metabolites (metabolomics) in a given organism. This “omics” revolution required dealing with increasing amounts of data, and new algorithms and computational approaches had to be developed. The easiness with which yeast cells can be grown and manipulated, combined with the already significant knowledge of yeast life-style, genes and proteins, made yeast (mainly *Saccharomyces cerevisiae*) a natural choice of organism in which to ask genome-wide questions. Technically, the new discipline of Systems Biology was driven by the sophisticated genetics of yeast, which allowed the construction of mutant collections, fusion protein collections, and many other tools. These, in turn, quickly became the basis for additional new genome-wide technologies 77. The Systems Biology revolution is still ongoing, and a communal effort is being made to map, for example, all the genetic and physical interactions in the yeast cells. Interestingly, as time passes, the field as a whole is shifting from general questions about genome architecture and function, to more focused questions related to particular aspects of Biology, which are analyzed with a mixture of Systems and Molecular Biology. Here we summarize the efforts directed at studying the biology of telomeres at a system-level.

## GENOME-WIDE STUDIES OF TELOMERE BIOLOGY

Telomere Biology is greatly benefitting from the new all-encompassing approach, which greatly enlarged our knowledge about the genes involved in telomere biology and their regulation. The yeast genome has close to 6000 recognized genes. A collection of 4700 mutants was constructed by systematically deleting each individual non-essential gene in yeast (non-essential yeast mutant collection, 78). This collection was later complemented by two additional mutants libraries encompassing all the essential genes (yeast has ~1300 essential genes): either hypomorphic 79 or temperature-sensitive alleles 80 of each of the essential genes were created. In addition to allowing systematic crosses of all possible yeast mutants with a particular mutation/gene construct/reporter 81, these mutant collections allow researchers to carry out systematic mutant screens even if the phenotype of interest is not selectable. Once a biochemical assay (or a microscopic phenotype) is available, it can be applied in a systematic fashion to each member of the deletion collections, searching systematically for mutant strains defective for the particular assay. Even complex assays that take hours to carry out, or microscopic phenotypes that require complex and slow measurements can be performed in the whole mutant collections in batches, so that the whole collection can be screened in a matter of months.

Three publications reported the systematic screening of the mutant collections, looking for those mutants that affect telomere length (*t*elomere *l*ength *m*aintenance or *tlm* mutants). DNA was extracted from each individual yeast strain and telomere length was measured by Southern blot, using as probes telomeric repeats that hybridize to the terminal restriction fragment 82,83,84. Together, these papers identified ~400 genes affecting telomere length (either shorter or longer than the wild type). To understand the strength of this approach, it suffices to mention that only 30 or so genes were known to affect telomere length at the time the screens were carried out 82. This list of genes underscores the central role played by telomere biology in the yeast life cycle, as ~7% of the genome affects telomere length. Moreover, it also demonstrates the complexity of the challenge: telomere length is determined by mechanisms that elongate (telomerase) or shorten (nucleases) telomeres (each of which may be positively and negatively regulated). Mutation in any of the *TLM* genes changes the final telomere size; this means that each of the 400 genes participates in determining the equilibrium between the two types of activity. It is remarkable to observe that in each genetic background (e.g., S288c or W303) wild type cells exhibit always telomeres of the same size; thus, in the tug-of-war between elongating and shortening mechanisms, the equilibrium is always attained at the same telomere length. Thus, a very strong homeostatic mechanism is at play to ensure the exact telomere size for each cell (Figure 2). The genes uncovered in these screens, as expected, include those affecting DNA and chromatin metabolism, but almost all functions in the cell are also represented, including RNA and protein synthesis, traffic and modification, metabolic pathways, mitochondrial functions, etc. The challenge ahead, of course, is to determine how all these genes impinge on the telomere length determination.

Up to now we have extoled the advantages of the genome-wide approach. One should, however, also take into consideration the fact that, by dealing with thousands of genes, there will always be inevitable errors. Despite claiming “completeness”, the mutant libraries are not always complete, as some mutants either fail to grow, accumulate suppressors, or exhibit aneuploidy and heterogeneous behavior. Moreover, one can also expect a number of handling errors. Thus, it is always advisable to confirm the identity of the mutant knockout by PCR, and to complement the phenotype of the mutant by introducing the wild type gene. Interestingly, attempts to carry out this type of validation uncovered an interesting phenomenon: in about 10% of the yeast deletion mutants, the phenotype observed is due to the effect that the deletion has had on the gene’s neighbor, rather than to the lack of function of the deleted gene. One can complement the phenotype of a strain deleted for the A gene with a plasmid carrying B, its neighboring gene, but not by a plasmid carrying the A gene itself 85.

One should thus regard results obtained in a genome-wide approach as candidates for validation, rather than as final results. The need to handle hundreds, and sometimes thousands of strains in parallel, does not always allow the detection of subtle differences, and may sometimes lead to contradictory results that can be resolved only by careful control experiments (e.g., 86 and 87).

The availability of a near-complete list of *TLM* genes opens the door for new modes of exploration of telomere biology. A simple approach is to analyze, using Molecular Biology approaches, the mechanisms affecting telomere length in a certain subset of *tlm* mutants. For example, Rog *et al.* took this approach to explore the role of vacuolar protein sorting (VPS) proteins, in charge of vacuolar traffic in the cell, in telomere length maintenance. The list of *TLM *genes is highly enriched for those encoding VPS proteins. The authors identified the pathway as being dependent on telomerase and Ku, but independent of Tel1 and Rif2 88.

As mentioned above, large amounts of information are being gathered about different aspects of yeast biology. Using computational approaches to take advantage of this rich resource, network models of the telomere biology have been established, allowing their study.

For example, Schahar *et al.* used network theory to integrate the list of TLM genes with a large protein-protein interaction database, thus rigorously charting the cellular subnetwork underlying the functions investigated. This method identified pathways connecting TLM gene functions to telomere length maintenance, and, at the same time, expanded the list of TLM genes by identifying nodes in the network that were predicted to participate in the information flow. These mutants either affected essential functions and were not included in the collections, or were not identified in the original screens for technical reasons. The authors experimentally validated some of these predictions, demonstrating that the method developed is remarkably accurate. This analysis, for example, identified the KEOPS complex (identified in parallel by two other groups 89,90), and also uncovered a link between the COMPASS histone H3 K4 methylation and the ESCRT vacuolar transport 91.

The telomere length dataset was further used to calibrate the methodology used for charting pathways within networks. A fundamental challenge in network analysis is to chart out the protein pathways that underlie a particular system. Previous approaches to the problem have either employed a local optimization criterion, aiming to infer each pathway independently, or a global criterion, searching for the overall most parsimonious subnetwork. Yosef and co-workers studied the trade-off between the two approaches and developed a new intermediary scheme that provides explicit control over it. This intermediary approach provided the most plausible solutions, which were then validated using molecular genetics approaches. This analysis identified a yet unappreciated link between proteasome activity, DNA repair mechanisms and telomere length maintenance 92.

## SECONDARY SCREENS OF *tlm* MUTANTS

Secondary screens were also carried out on the *tlm* mutant collection. In one of these, *TLC1* RNA levels were measured in all *tlm* mutants, and 24 of them were found to affect telomere length via their effect on *TLC1* levels 93. These results identify the mechanism of action of some Tlm proteins, and suggest that the level of telomerase RNA may be limiting in telomere length maintenance. The list of mutants included four subunits of Paf1C (Polymerase II-associated factor complex). While Paf1C had been implicated in the transcription of both polyadenylated and nonpolyadenylated RNAs, it had not been associated previously with the noncoding telomerase RNA. Telomere length in Paf1C mutant strains can be compensated by TLC1 overexpression, suggesting that telomerase RNA is a critical direct or indirect Paf1C target 93.

A second screen explored the effect of starvation on telomere length 94. Starved cells, or those exposed to the TORC1 inhibitor rapamycin, respond by dramatically shortening their telomeres. Assuming that any signal that affects telomere length must proceed via *TLM*-controlled pathways, Ungar and co-workers screened the *tlm* mutants for those that do not respond to the starvation signals. They found that most *tlm *mutants responded normally to starvation signals; *yku70 *and *yku80* mutants, however, fail to respond to rapamycin, indicating that the Ku heterodimer plays a central role in the starvation response. The authors dissected the pathway involved in signal transduction, and found that the TORC1 complex controls the localization of the Gln3 and Gat1 transcription factors. When cells are starved, these factors enter the nucleus and reduce the levels of Ku protein, thus affecting telomere length. This finding is particularly interesting in light of studies suggesting that calorie restriction may lengthen lifespan, whereas telomere attrition leads to cellular senescence 94. This apparent paradox needs further attention, and will no doubt be the subject of future research.

In another study, that followed the response of yeast telomeres to environmental stimuli, it was found that exposure to ethanol elongates telomeres, whereas caffeine and high temperature reduce telomere length 34. Again, the authors systematically tested *tlm *mutants looking for those that did not respond or were over-responders to the external signal. They found that different environmental cues seem to act via different pathways; remarkably, ethanol and caffeine required both the activity of Rif1, the Rap1 binding telomeric protein. Rif1 thus is essential for the transmission of both elongating and shortening signals to the telomeres. Interestingly, *rif2 *strains, as well as mutants of the MRX/Tel1 pathway (analogous to the mammalian MRN/ATM pathway) exhibited a stronger-than-expected response to ethanol, which causes telomere elongation, indicating that these proteins have a role in preventing length-independent elongation of telomeres. This result is particularly interesting in light of the fact that the MRX/Tel1 pathway is important for telomere elongation, and is believed to play a pivotal role in choosing the shortest telomeres for elongation, thus maintaining the telomere length homeostasis 72. The work of Romano and co-workers also identified other genes involved in the response to external stimuli: for example, the Nonsense Mediated Decay (NMD) pathway, which regulates the half-life of many RNA molecules in the cell, is important for the response to ethanol. The authors found that this is due to regulation of STN1 mRNA by the NMD; indeed, it is enough to overexpress Stn1 and Ten1 in wild type cells to prevent elongation by ethanol (the phenotype observed in *nmd* mutants), demonstrating that the CST plays a positive role in the response. The vacuolar transport pathway and the chaperone Hsp104 were also found to play important roles in the presence of ethanol. In contrast, caffeine was shown to affect telomere length by controlling the activity of the ATM and ATR-like kinases, Tel1 and Mec1. Surprisingly, all *tlm *mutants responded to high temperature, suggesting that the response to high temperature is due to an intrinsic inactivation of the telomere machinery itself, rather than a defect in the transmission of a signal 34.

Finally, Jin-Qiu Zhou and colleagues explored the *tlm* collection looking for mutants that affect the survival pathways in the absence of telomerase activity 95. Previous work has shown that in the absence of either telomerase’s catalytic subunit, its Est co-factors or its template RNA, cells senesce due to constant telomere shortening. From a population of senescing cells, rare survivors arise by recombination-based mechanisms, also known as ALT (Alternative Lengthening of Telomeres)^96^. Survivors are usually of two types: Type I survivors amplify internal subtelomeric repeats, but have telomeric repeats of normal length, whereas Type II have long telomeric repeats. Whereas Type I survivors are more common, they grow slowly, and in liquid cultures are usually overgrown by Type II cells 96,97. Hu and co-workers carried their secondary screen of *tlm *mutants by knocking out the *TLC1* gene, encoding telomerase RNA, in 280 *tlm *mutants. They then monitored the patterns of senescence and survival. New functional roles were found for 10 genes that affect the emerging ratio of Type I versus Type II survivors and 22 genes that are required for Type II survivor generation. For example, the Pif1 helicase and the INO80 chromatin remodeling complex reduced the frequency of Type I survivors, whereas the KEOPS complex (see below) was shown to be required for Type II recombination 95.

## ADDITIONAL GENOME-WIDE SCREENS FOR TELOMERE-RELATED FUNCTIONS

### 1) Functions important upon telomere uncapping:

Binding of the Cdc13 to telomeric ssDNA repeats is essential for telomere protection. Upon transfer of cells carrying the temperature-sensitive *cdc13-1* allele to the restrictive temperature, for example, telomeres become “uncapped” and extensive resection of telomeres leads to senescence, cell cycle arrest and cell death. The Lydall lab conducted a systematic screen for mutants that affect growth of a *cdc13-1* allele. By crossing a *cdc13-1* mutant with the whole non-essential deletion collection, they identified 369 gene deletions that affected growth of *cdc13-1* mutants at several temperatures. The mutants could be divided in eight phenotypic categories. The results included many of the checkpoint-affecting genes expected, but also genes in a variety of unexpected categories, such as RNA metabolism and phosphate and iron homeostasis. In addition, the screen identified a number of genes of previously unknown function renamed RTC (restriction of telomere capping) or MTC (maintenance of telomere capping) 89,98. The KEOPS (*k*inase, *e*ndopeptidase and *o*ther *p*roteins of *s*mall size) complex is a good example of a group of proteins with a central role in telomere biology identified in genome-wide screens. Mutations in KEOPS members affect telomere length and capping, and were also found in the screen for mutants that affect the kinetics of survivor appearance 89,95. This complex is composed of proteins, some of which are conserved in the three domains of life, and some of which can be found only in fungi: Kae1, a putative endopeptidase with an unknown role, is absolutely conserved in Archea, Bacteria and Eukarya; Bud32, is a serine/threonine kinase with a wide distribution in the tree of life; Pcc1, the yeast homolog of the two human cancer-testis antigens that are specifically expressed in different tumors but also in normal testes and ovaries 90, is also widely distributed; Gon7 (also referred to as Pcc2) is a small protein with no known functional domains, found only in fungi; and Cgi121, whose human homolog binds the human Bud32, also known as the p53-related protein kinase 99. Mutations in KEOPS components partially suppress the temperature-sensitivity of *cdc13-1*, caused by telomere uncapping. Interestingly, further biochemical work identified the main role of KEOPS as the N6-threonylcarbamoylation (t(6)A) of tRNA, an essential modification required for translational fidelity by the ribosome 100.

This capping mutant screen was extended, by systematically looking for suppressors or enhancers of the *yku70∆* mutation, and comparing them to the results obtained for *cdc13-1*. The authors then developed a sophisticated analysis, named QFA (Quantitative Fitness Analysis), which allowed them to dissect the genetic interactions of both capping complexes, CST and Ku. The response to telomere uncapping was shown to be genetically complex, with many genes involved in a variety of processes affecting the outcome. Many genes had differential effects: for example, inactivating the Nonsense Mediated Decay (NMD) pathway, which affects half-life of mRNAs in the cells, led to suppression of *cdc13-1* but enhanced the phenotype of *yku70∆*. The authors showed that this effect is Stn1 dependent, again stressing the pivotal role of the CST in regulating telomere biology 101.

### 2) Genes important for telomere three-dimensional configuration:

An additional genome-wide screen looked for mutants affecting the telomere three-dimensional configuration. In mammalian cells telomeric DNA forms a loop that allows the ssDNA end to invade telomeric dsDNA forming a Holliday Junction, in a structure that protects the ssDNA end and is called a T-loop 2. Due to the small size of yeast telomeres, it has been impossible to visualize T-loops in yeast, as has been done in mammals. Instead, functional assays have been used. For example, a construct carrying a TATA-less galactose-inducible UAS (*u*pstream *a*ctivating *s*equence) downstream of the *URA3* gene is able to transcribe the *URA3* gene only at telomeres, where it could fold back on itself, but not at other locations in the genome 3. The Luke group took advantage of this fact to carry out a genome-wide screen for mutants affecting telomere fold-back, looking for those that were unable to express the *URA3* construct and could thus grow on medium containing galactose and 5-FOA (which is lethal when *URA3* is expressed). This screen identified 112 genes that disrupt looping when mutated 3. Among various biological processes uncovered, lysine deacetylation was found to be essential for the fold-back through Rif2-dependent recruitment of the Rpd3L complex to telomeres. Absence of Rpd3 function generates increased susceptibility to nucleolytic degradation and the initiation of premature senescence, suggesting a protective role for Rpd3 deacetylation activity 3. Thus, this work identifies a major mechanism of end protection, together with the way it is recruited to telomeres.

### 3) Genes affecting the Tel1 (ATM) pathway:

A screen for enhancers of the MMS sensitivity of *tel1∆* mutants uncovered a small number (13) of genes. These included Yku70, members of the 9-1-1 pathway, the CCR4-NOT deadenylase complex, nuclear pore components and several histone deacetylases. Most of these mutants caused the MMS sensitivity due to their effects on telomeres 102. Interestingly, this report also suggests that shortened telomeres confer sensitivity to DNA damaging agents, such as methyl methanesulfonate (MMS).

### 4) Genes affecting senescence:

The *tlm *collection was screened by Hu and co-workers, looking for mutants affecting survivor appearance in the absence of telomerase activity 95(see above). A genome-wide screen 87 examined, in a systematic fashion, the kinetics of senescence, by crossing the *est1∆* mutation (lacking an essential telomerase recruitment factor) to the whole nonessential mutant collection. As expected, the vast majority of gene deletions showed no strong effects on the entry into/exit from senescence. However, ~200 gene deletions (among them the well-characterized *rad52∆* mutant) accelerated entry into senescence, and such cells often could not recover growth. A smaller number of strains (among them *rif1∆*) accelerated both entry into senescence and subsequent recovery 87. Interestingly, the mutants that accelerated senescence tended to also have negative genetic interactions with *yku70∆*, but were neutral when combined with *cdc13-*1, as determined in the genome-wide screen described above 101.

Although the Systems Biology revolution is only at its infancy, we can summarize at this stage that the genome-wide studies have vastly extended our view of telomere biology. The number of cellular processes affecting various aspects of telomere integrity, replication, length regulation and structure is remarkable. Most of the genes uncovered in these screens are evolutionarily conserved, and likely to act similarly in other organisms, including humans. A better understanding of the mechanisms regulating telomere biology will have significant medical implications, especially in the fields of aging and cancer.
